# Supplementary material for: How many individuals share a mitochondrial genome?
Source: PLoS Genet. 2018 Nov 1;14(11):e1007774. doi: 10.1371/journal.pgen.1007774 (PMC6233927; doi:10.1371/journal.pgen.1007774)
Supplement: S1 Table — Key quantiles of the distributions shown in Fig 2 for the mutation scheme of Översti [13], and for the 300K constant demographic scenario. (PDF) [file pgen.1007774.s001.pdf]

Quantile	50%	95%	99%
Unconditional	149	675	1,085
n = 100 / m = 0	138	624	989
n = 1,000 / m = 0	86	380	585
n = 10,000 / m = 0	18	79	121
n = 100 / m = 1	351	1,030	1,469
n = 1,000 / m = 1	211	605	859
n = 10,000 / m = 1	44	124	173
n = 100 / m = 2	568	1,360	1,573
n = 1,000 / m = 2	343	816	1,103
n = 10,000 / m = 2	71	165	221
n = 1,000 / m = 5	745	1,418	1,573
n = 10,000 / m = 5	148	275	345
n = 10,000 / m = 10	280	450	533
