# Supplementary material for: How many individuals share a mitochondrial genome?
Source: PLoS Genet. 2018 Nov 1;14(11):e1007774. doi: 10.1371/journal.pgen.1007774 (PMC6233927; doi:10.1371/journal.pgen.1007774)
Supplement: S2 Table — Key quantiles of the distributions shown in Fig 2 for the mutation scheme of Översti [13], and for the 1.2M growth demographic scenario. (PDF) [file pgen.1007774.s002.pdf]

Quantile	50%	95%	99%
Unconditional	295	2,869	5,603
n = 100 / m = 0	268	2,524	4,655
n = 1,000 / m = 0	161	1,134	2,126
n = 10,000 / m = 0	46	231	375
n = 100 / m = 1	1,548	6,042	9,108
n = 1,000 / m = 1	661	2,556	3,665
n = 10,000 / m = 1	130	406	588
n = 100 / m = 2	3,246	9,108	10,561
n = 1,000 / m = 2	1,372	3,683	5,340
n = 10,000 / m = 2	223	569	782
n = 1,000 / m = 5	3,567	7,168	9,177
n = 10,000 / m = 5	534	1,038	1,302
n = 10,000 / m = 10	1,084	1,762	2,140
