# Supplementary material for: How many individuals share a mitochondrial genome?
Source: PLoS Genet. 2018 Nov 1;14(11):e1007774. doi: 10.1371/journal.pgen.1007774 (PMC6233927; doi:10.1371/journal.pgen.1007774)
Supplement: S3 Table — Key quantiles of the distributions shown in Fig 2 for the mutation scheme of Rieux [14], and for the 1.2M constant demographic scenario. (PDF) [file pgen.1007774.s003.pdf]

Quantile	50%	95%	99%
Unconditional	177	761	1,148
n = 100 / m = 0	174	744	1,114
n = 1,000 / m = 0	146	627	956
n = 10,000 / m = 0	56	244	375
n = 100 / m = 1	416	1,154	1,627
n = 1,000 / m = 1	352	981	1,364
n = 10,000 / m = 1	137	386	543
n = 100 / m = 2	658	1,528	2,136
n = 1,000 / m = 2	558	1,297	1,725
n = 10,000 / m = 2	219	514	686
n = 1,000 / m = 5	1,154	2,151	2,293
n = 10,000 / m = 5	463	856	1,061
n = 10,000 / m = 10	862	1,364	1,639
