# Supplementary material for: How many individuals share a mitochondrial genome?
Source: PLoS Genet. 2018 Nov 1;14(11):e1007774. doi: 10.1371/journal.pgen.1007774 (PMC6233927; doi:10.1371/journal.pgen.1007774)
Supplement: S4 Table — Key quantiles of the distributions shown in Fig 2 for the mutation scheme of Rieux [14], and for the 300K constant demographic scenario. (PDF) [file pgen.1007774.s004.pdf]

Quantile	50%	95%	99%
Unconditional	193	859	1,293
n = 100 / m = 0	176	784	1,190
n = 1,000 / m = 0	99	432	676
n = 10,000 / m = 0	18	81	124
n = 100 / m = 1	440	1,222	1,605
n = 1,000 / m = 1	242	702	982
n = 10,000 / m = 1	45	128	179
n = 100 / m = 2	704	1,517	1,827
n = 1,000 / m = 2	391	932	1,228
n = 10,000 / m = 2	73	169	226
n = 1,000 / m = 5	836	1,507	1,818
n = 10,000 / m = 5	151	285	355
n = 10,000 / m = 10	290	458	545
