# Supplementary material for: How many individuals share a mitochondrial genome?
Source: PLoS Genet. 2018 Nov 1;14(11):e1007774. doi: 10.1371/journal.pgen.1007774 (PMC6233927; doi:10.1371/journal.pgen.1007774)
Supplement: S5 Table — Key quantiles of the distributions shown in Fig 2 for the mutation scheme of Rieux [14], and for the 1.2M growth demographic scenario. (PDF) [file pgen.1007774.s005.pdf]

Quantile	50%	95%	99%
Unconditional	387	3,835	7,361
n = 100 / m = 0	339	3,242	5,662
n = 1,000 / m = 0	182	1,291	2,342
n = 10,000 / m = 0	47	237	386
n = 100 / m = 1	2,004	7,697	11,463
n = 1,000 / m = 1	756	2,875	4,164
n = 10,000 / m = 1	133	415	608
n = 100 / m = 2	4,027	11,275	14,221
n = 1,000 / m = 2	1,544	4,133	5,579
n = 10,000 / m = 2	228	586	806
n = 1,000 / m = 5	3,926	7,799	9,608
n = 10,000 / m = 5	552	1,057	1,332
n = 10,000 / m = 10	1,095	1,779	2,134
