# Supplementary figures and images for: How many individuals share a mitochondrial genome?
Source: PLoS Genet. 2018 Nov 1;14(11):e1007774. doi: 10.1371/journal.pgen.1007774 (PMC6233927; doi:10.1371/journal.pgen.1007774)

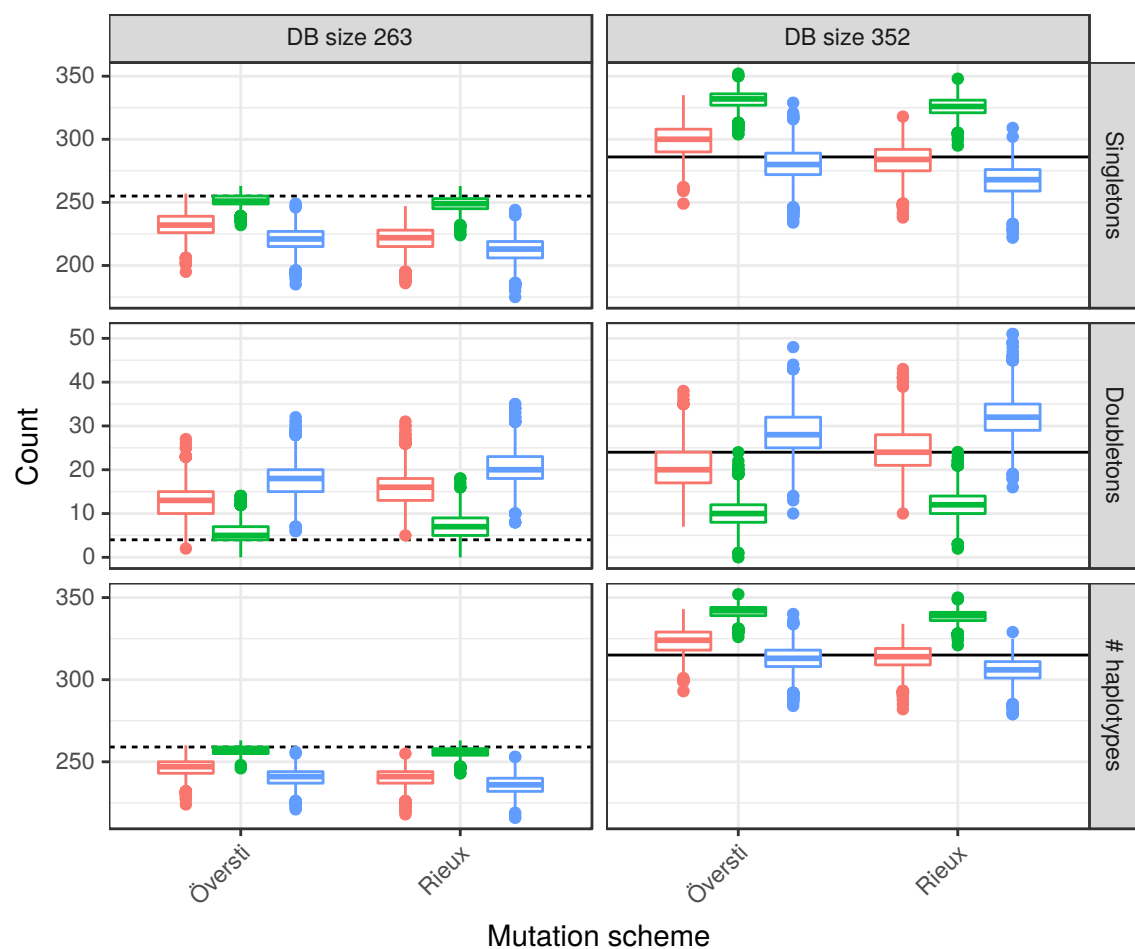

Source

— Derenko et al. (2013)

---- Just et al. (2015)

Population size

1.2M growth 300K const.

1.2M const.

Supplement: S1 Fig — The distribution of the numbers of singletons, doubletons and distinct haplotypes in 2,500 random databases of sizes 263 and 351 obtained under our three demographic and two mutation models. The horizontal reference lines are from [15, 16]. [16] does not provide number of singletons and doubletons, but these numbers (286 and 24, respectively) were obtained directly from the authors. The boxes extend from 25% to 75% quantiles, and the median is indicated with a line segment. The whiskers are constructed as 1.5 times the interquartile range and the outliers show the observations outside the whiskers. (PDF) [file pgen.1007774.s006.pdf]
